# Veterinary Medical Association of New Jersey

**Published:** 1895-11

**Authors:** S. Lockwood

**Affiliations:** Secretary


### VETERINARY MEDICAL ASSOCIATION OF NEW JERSEY.

There was an unusually large meeting held in Achtel Stetter's, at 842 Broad street, Newark, on Thursday, October 10, 1895. It was called to order shortly after 11 o'clock by the President, Dr. J. W. Hawk, of Newark; besides whom there were present Drs. J. Gerth and Runge, of Newark; M. M. Stage, of Dover; R. O. Hasbrouck, of Passaic; Hugh Exton, of Washington; B. F. King, of Little Silver; William H. Lawes, of Red Bank; William Gall, of Mattawan; P. Brown, of Windsor; L. P. Hurley, of Hopewell; E. Britton, of Long Branch; S. Lockwood, of Woodbridge; and J. W. Stickler, M. D., of East Orange.

President Hawk suspended the regular order of business to permit the Board of Censors to examine two candidates. Both were subsequently elected to membership by a unanimous vote. President Hawk then made an address, in which he referred to the so-called anthrax which has been scaring the people of the State during the last few months, and declared his belief that there was no anthrax in New Jersey, as there had never been an official report on the matter from a veterinarian.

The Secretary reported Dr. A. D. Edwards, of Atlantic Highlands, as an applicant for membership.

Dr. Dustan read a paper on the organization and growth of the Association.

Dr. Runge reported on the result of a trip to Cumberland County in search of anthrax, and had specimens and a microscope on hand for the benefit of the members.

After Dr. Gerth had read an interesting paper on meat inspection, the meeting adjourned at 2 o'clock in the afternoon for dinner.

It was reconvened at 3.40 o'clock and Dr. Gerth's paper was discussed at length by Drs. Runge, Dustan, Everett, Hawk, and King. On motion of Dr. Hawk a committee of three was appointed to investigate the manner in which the money was spent which was appropriated by the State for the stamping out of tuberculosis in New Jersey, by a commission of the State Agricultural Board.

Drs. L. P. Hurley, W. B. E. Miller, and William Gall were named as the essayists for the next meeting.

The meeting adjourned to come together in April.

S. LOCKWOOD,
Secretary.

---

THE meeting of the California State Veterinary Medical Association, on the 10th of September, was not a very successful one, only local veterinarians being in attendance.

